# Explainable machine learning for real-time deterioration alert prediction to guide pre-emptive treatment

**DOI:** 10.1038/s41598-022-15877-1

**Published:** 2022-07-11

**Authors:** Aida Brankovic, Hamed Hassanzadeh, Norm Good, Kay Mann, Sankalp Khanna, Ahmad Abdel-Hafez, David Cook

**Affiliations:** 1 CSIRO Australian e-Health Research Centre, Brisbane, QLD 4029 Australia; 2 Metro South Health, Brisbane, QLD 4102 Australia; 3 Intensive Care Unit, Princess Alexandra Hospital, Brisbane, QLD 4102 Australia

**Keywords:** Translational research, Health care, Risk factors, Information theory and computation, Biomedical engineering, Software

## Abstract

The Electronic Medical Record (EMR) provides an opportunity to manage patient care efficiently and accurately. This includes clinical decision support tools for the timely identification of adverse events or acute illnesses preceded by deterioration. This paper presents a machine learning-driven tool developed using real-time EMR data for identifying patients at high risk of reaching critical conditions that may demand immediate interventions. This tool provides a pre-emptive solution that can help busy clinicians to prioritize their efforts while evaluating the individual patient risk of deterioration. The tool also provides visualized explanation of the main contributing factors to its decisions, which can guide the choice of intervention. When applied to a test cohort of 18,648 patient records, the tool achieved 100% sensitivity for prediction windows 2–8 h in advance for patients that were identified at 95%, 85% and 70% risk of deterioration.

## Introduction

Globally, hospitals have begun the transition to becoming fully digital environments, prompting a revolutionary change in the way healthcare is delivered and patients are monitored. Capturing patient data in Electronic Medical Records (EMR) presents an opportunity to manage patient care efficiently and accurately. One example is to use digital tools to monitor patient vital signs and subsequent algorithms to automate and improve the detection of clinical deterioration in a ward setting, generally associated with adverse events such as unplanned transfer to an Intensive Care Unit (ICU), cardiac arrest or death^1–3^.

Current hospital workflows rely on the use of clinical scoring and rule-based track and trigger tools such as the Australian Between the Flags (BTF), National Early Warning Score (NEWS), and Queensland Adult Deterioration Detection System (Q-ADDS) to alert against the risk of clinical deterioration^1,4,5^. These tools have evolved as a response to clinical need and are somewhat lacking in methodological rigor and validation^4,6^. Other limitations of track and trigger systems are discussed in^7–9^. Nonetheless, they are an important part of maintaining patient safety standards and are used to trigger escalation of care including attendance of a rapid response team (RRT) or medical emergency team with the intention of avoiding preventable deterioration. Various statistical modelling and machine learning based approaches have recently been proposed as replacements and/or improvements to these existing clinical track and trigger tools^2,3,7,10–12^. These statistical or machine learning based approaches, however, present two problems that are yet to be overcome by existing efforts in this space. Firstly, the use of historical patient data from hospitals where the clinical workflow already includes a particular track and trigger protocol confounds the subsequent development of alternate criteria-based algorithms. This is because the existing protocols would have resulted in alerts that would have been raised and acted upon by the clinical teams, distorting the true relationship between vital sign derangement and any potential adverse outcome. Identifying such actions in the EMR and accounting for them in risk prediction algorithm development is also not straightforward. Secondly, these approaches are hard to trial and validate in settings where the use of existing systems like BTF, NEWS and Q-ADDS is already mandated. Recent reviews suggest that there is limited evidence of uptake and impact from such existing algorithms^1^.

We have adopted an approach to “predict the alert”, and aim to identify clinical deterioration earlier, before activation of the rapid or emergency response protocols currently in use. We have developed and validated a machine learning algorithm that predicts the current mandated track and trigger protocol before the escalation criteria is triggered. A pre-emptive, rather than reactive, process therefore allows the clinical response team additional time to intervene and prevent clinical deterioration within existing workflows. Addressing clinical patient deterioration earlier, before activation of existing (and possibly different) rapid or emergency response and intervention also helps mitigate the confounding effects from the treatment and/or intervention that can be hard to identify in the data. Secondly, this approach allowed for the use of model explainability to provide insight into the likely causes of predicted deterioration events to support clinical decision making when responding to alerts. This explainability provides a guide as to the pathway and likely observations that will allow recognition of clinical deterioration, and hence infer the underlying mechanisms and inform treatment options.

To the best of our knowledge, this is a novel approach aimed at anticipating deterioration early by pre-empting alerts from existing track and trigger systems in clinical practice, rather than predicting death, cardiac arrest or ICU transfer.

To achieve these aims, we present the development and validation of an explainable real-time machine learning (ML) algorithm denoted as the Encounter-based Forest-like (EF) ensemble model for Predicting the Risk of Deterioration (PRoD) of a patient within a ward setting. The EF model was designed to employ Australian acute care EMR data in real-time and to provide an explainable prediction of a deterioration risk alert being triggered up to 8 hours before the actual event. We also introduce a novel ensemble model that used hospital encounter-based sampling and discuss how this approach was better suited to the class of problems being solved. The BTF alert was employed as the predicted trigger protocol for this study given its mandated use within the Princess Alexandra Hospital (PAH), a large Tertiary hospital in Queensland, Australia. Based on BTF, safe or normal value ranges are defined for each vital sign. Patients’ vital signs charts are tracked over time to check whether they are in normal or abnormal ranges. A yellow or red flag is triggered every time vital signs derange or exceeds threshold of normal ^13^. Models were trained on vital signs data collected from $n = 48,298$ patients (75, 846 hospital encounters) admitted to a hospital bed for acute care between January 2016 and December 2018 at the PAH. A historical window of 24 h was used to anticipate the stability of a patient’s condition in the following 2, 4, 6 and 8 h. Predictive performance of all trained models was evaluated retrospectively on a test dataset consisting of $n = 14,387$ patients (18, 683 encounters) admitted to the same hospital between 1 January 2019 and 30 September 2019 for prediction windows of 2, 4, 6 and 8 h ahead. Though developed for the BTF protocol, the workflow and model process of the EF is easily re-adjustable to fit any other early warning system.

## Results

### Data characteristics

**Figure 1:**
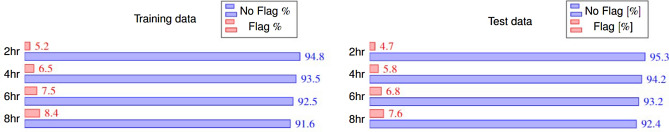
Class distribution per alerting horizon in train and test data partitions.

Baseline characteristics of the considered cohort are reported in Table 1. The train and test datasets consisted of 2,418,646 and 818,955 records, respectively. Mean age was 59 years (STD 17 years) with 62% of patients being male. The proportion of BTF red flags varied between 5 and 8% for the 2 to 8 h prediction windows respectively across the train and test datasets (Fig. 1). As shown, just 5% of patients triggered red flag within 2 h prediction window, 6% within 4 h, 7% within 6 h and 8% within 8 h ahead of the red flag event.

**Table 1:** Vital signs, demographics data and patient consciousness for analyzed cohort represented by mean and standard deviation (STD).

Variable name	Variable range	Train	Test	p value*	SMD**
		2016–2018	Jan 2019–Sept 2019		
		(n = 2,418,646)	(n = 818,955)		
Systolic blood pressure (SBP) *mmHg*, mean(STD)	[0, 300]	126.69 (21.16)	126.50 (20.92)	< 0.001	0.009
Diastolic blood pressure (DBP) *mmHg*, mean(STD)	[0, 250]	73.14 (11.12)	73.24 (11.24)	< 0.001	0.009
Mean arterial pressure (MAP) *mmHg*, mean(STD)	[20, 261]	91.08 (12.98)	91.07 (12.92)	0.662	0.001
Heart rate $min^{-1}$, mean(STD)	[0, 200]	79.31 (15.92)	78.93 (15.76)	< 0.001	0.024
Temperature $\deg C$, mean(STD)	[30, 42.2]	36.70 (0.40)	36.70 (0.39)	< 0.001	0.018
Respiratory rate $min^{-1}$, mean(STD)	[0, 60]	17.37 (2.70)	17.31 (2.57)	< 0.001	0.022
Oxygen saturation (SpO2) $\%$, mean(STD)	[50, 100]	96.52 (2.33)	96.55 (2.28)	< 0.001	0.013
Level of consciousness (AVPU) Count Yes ($\%$)	Yes/No	1,139,246 (47.1)	604,482 (73.8)	< 0.001	0.568
Oxygen flow rate measurement count yes ($\%$)	Yes/No	870,033 (36.0)	275,136 (33.6)	< 0.001	0.05
Sex male($\%$)	Male/female	1,494,810 (61.8)	508,377 (62.1)	< 0.001	0.006
Age *years*, mean(STD)	[1, 106]	59.89 (17.23)	59.76 (17.20)	< 0.001	0.008
Length of stay *min*, mean(STD)	[0, 1,280,016]	13,468.90 (27828.52)	12,188.85 (22409.22)	< 0.001	0.051

*Calculated for training and test partition using two-sample t tests for normally distributed for descriptive purpose.

**Standardised mean difference (SMD).

### Predictive performance

Logistic regression (LR), Decision Tree (DT), Random forest (RF), Boosted trees (XGB), and a novel Encounter-based Forest-like (EF) ensemble model were trained to predict the likelihood of triggering a BTF red flag 2, 4, 6 and 8 h before an actual event. The final values of the hyperparameters for the aforementioned algorithms are reported in Supplementary Table S1. The performance of the trained models was evaluated on the test data (January–September 2019). Whilst a range of metrics, including Area Under the Receiver Operating Curve (AUC-ROC), Area under the Precision-Recall Curve (AUC-PRC), Positive Predictive Value (PPV), Negative Predictive Values (NPV), Recall and F1 score, were employed and reported, AUC-PRC was used as the guiding criteria for hyperparameter tuning and model selection given the class imbalance in the dataset. The key findings are reported in this section.

#### Tree-based methods superior to logistic regression

Average AUC-PRC performances and the corresponding $95\%$ confidence intervals (CIs) of individual LR, DT, RF and XGB models, trained on 1000 bootstrapped sub-samples for prediction horizons of 2 to 8 h were $0.405(0.404,0.405)-0.387(0.386,388)$, $0.562(0.561, 0.562)-0.459(0.459, 0.46)$, $0.632(0.632, 0.633)-0.536(0.536, 0.537)$ and $0.628(0.628{,} 0.629)-0.519$ (0.519, 0.52), respectively (Supplement, Table S2). While AUC-ROC and NPV performances were high and somewhat comparable across all models across the considered prediction windows, LR performed poorly on Precision and Recall, and consequently had a lower AUC-PRC score.

#### Comparable AUC-PRC for all tree-based methods, though the highest precision was obtained with EF model

AUC-PRC for EF and selected LR, DT, RF and XGB models across the considered prediction windows ranges were $0.6179(0.6177,0.6181)-0.5262(0.526,0.5263)$, $0.4048(0.4046,0.405)-0.3837(0.3836,0.3839)$, $0.5919(0.5917,0.5921)-0.4727(0.4726,0.4729)$, $0.6362(0.636,0.6364)-0.542(0.5418,0.5421)$, $0.631(0.6308,0.6312)-0.5222(0.522,0.5223)$, respectively (Supplement, Table S3). While Recall performance of EF was comparable to DT and slightly lower than XGB and RF, it offered significantly improved precision, especially where the prediction window was larger. At the 8 h prediction window, 12% higher precision than RF was performed whilst sacrificing recall performance by 6%, and 24% higher precision than XGB whilst sacrificing recall performance by 7%.

All performance metrics and corresponding CIs obtained with EF and selected DT, RF and XGB models on 1000 bootstrapped samples drawn with replacement from the test dataset across the considered prediction horizons are reported in Supplement, Table S3.

#### EF identifies correctly above 90% patients at highest risk across the time windows

High precision, comparable NPV and comparable AUC-PRC across the prediction horizons led to selection of EF as the best performing model and the candidate algorithm for any future trial in a clinical setting. To better understand it’s performance at identifying patients at high risk of triggering a BTF alert, precision and recall performance of this algorithm were evaluated specifically for the patient cohort that scored a Deterioration Risk Index 1, 2 and 3, representing patients in the top 5%, 6%-15% and 16%-30% risk groups respectively (Table 2). Precision of predicting the Risk Index 1 ranged from 92% to 95% across the prediction windows, and dropped to between 75% and 82% for Risk Index 3 group. Recall performance was 100% across these high-risk patient cohorts.

#### EF is well calibrated at high probabilities

**Figure 2:**
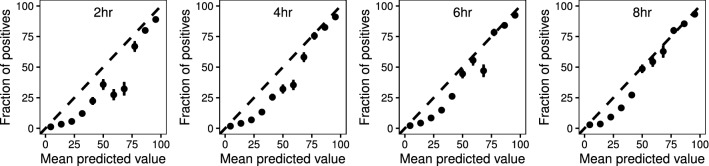
Calibration plots of EF for 2, 4, 6, and 8hr window.

Calibration plots (Fig. 2) show that ET calibrates well at high probabilities. For the 4, 6, and 8 h prediction windows the confidence intervals associated with the risk groups at highest risk cross the expected calibration curve indicating close to perfect calibration, hence provide statistical confidence in the obtained results. In contrast the model does not calibrate well around the lower probabilities, particularly for the 2 h prediction window however, this does not diminish the value of the results as the study aimed target patients who were at higher risk.

**Table 2:** EF model assessment for 2, 4, 6 and 8hr prediction window: Precision and recall computed for defined deterioration risk index.

Prediction window risk index	2 h		4 h		6 h		8 h	
	Precision	Recall	Precision	Recall	Precision	Recall	Precision	Recall
1 (Top 5 $\%$)	0.9218	1	0.9323	1	0.9408	1	0.9465	1
2 (Top 6–15 $\%$)	0.8241	1	0.8491	1	0.8690	1	0.8767	1
3 (Top 16–30 $\%$)	0.7531	1	0.7735	1	0.8130	1	0.8174	1

### Model and prediction explainability

**Figure 3:**
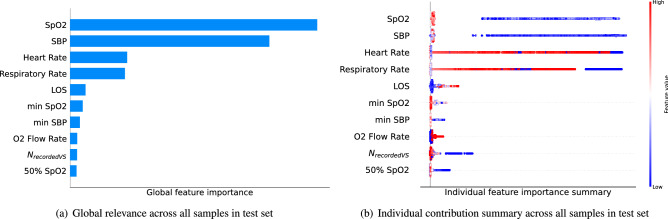
Output of the interpreter module showing the global variable importance (**a**) and the individual explanation summary (**b**) for the patients predicted to trigger the EWS in the next 4 h.

Being accountable for treatment mistakes, clinicians must be able to understand the underlying reasoning of models so they can trust the prediction^14^. Therefore, that transparency and explainability are an absolute necessity for the widespread introduction of ML/AI into clinical practice. Clifton et al.^15^ discuss instances where deterioration alerts may be raised when patients appear “normal”, and this potentially creates an overhead for the clinicians responding to the alert. Our interaction with clinicians on developing similar algorithms for use in clinical settings has also reinforced the need for predictions to be explainable. Our approach incorporates both, population level explainability and individual prediction-level explainability. An interpreter module was built during the training phase and to allow insight into the rationale behind the predicted output for an individual, i.e. patient-specific, and a population-based perspective. Fig. 3 shows the top 10 contributors for predicting a triggering of the BTF red flag from the population perspective. The variables in Fig. 3a and b are sorted in descending order in both plots in relation to their mean relevance computed as the mean absolute value of the Shapley values for each feature Each horizontal blue bar in Fig. 3a indicates the mean relevance of the variable for the considered population (i.e., a relative global importance). Figure 3b shows a summary of the all individual contributions that are colored based on variable value and sorted by the mean relevance.

**Figure 4:**
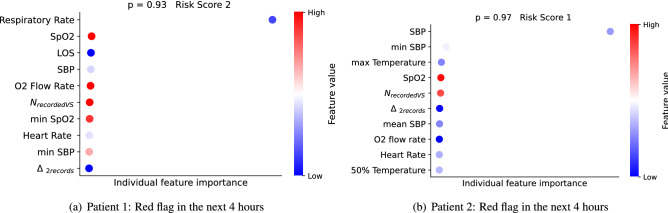
Interpreter output for two individual patients predicted to trigger the red flag in the next 4 h with the computed probabilities and the corresponding deterioration risk index.

Figure 4 shows the interpretations for two randomly selected samples corresponding to patients who were predicted to have a red flag event within 4 h. It displays information in three ways: (1) Top ten contributors to these particular patients that drive the evolving risk of emergency calls in the next 4 h are shown in descending order (top to bottom), (2) the colour of the dot for each feature indicates the value of the corresponding feature for that data sample, such that extremes of the color-bar (red and blue) represent the highest and lowest value of the corresponding feature respectively, (3) the distance from the vertical axis is determined by the Shapely value computed for that feature, i.e. the more distant point is the bigger is Shapely value and hence its relevance in the prediction. In Fig. 4a, low respiratory rate was the main contributor along with oxygen saturation, supplemental oxygen delivery and low systolic blood pressure. This would inform the attending clinician that the mode of risk was respiratory failure, characterized by low respiratory rate, such as would occur with opiate excess. In Fig. 4b, particularly low systolic pressure with low temperature, low heart rate and oxygenation were contributing. This scenario of phenotype would be indicative of sepsis or infection.

## Discussion

The aim of this study was to develop a prediction tool to identify patients that were at high risk of triggering a deterioration alert 2–8 h before it was actually triggered within the clinical setting. Our work predicts the BTF red-alert, this being the protocol of choice in a dataset from a large tertiary Hospital in Australia. A BTF red alert event occurs when a vital sign measurement falls outside the threshold of normal or moderate derangement.

We present a novel forest-like ensemble machine-learning algorithm, called an Encounter Forest (EF), which employs hospital encounter-level bootstrapping to avoid a bias towards sicker and longer staying patients, a DT architecture for each bootstrapped sub-sample, and bootstrap aggregation for obtaining the final probability predictions. This approach was compared to existing state of the art LR, DT, XGB and RF algorithms which were also trained on encounter-level bootstrapped sub-samples for fair comparison. Our evaluation showed that EF offers comparable or better performance than other models across a range of performance metrics, while offering significantly higher precision than other methods at the expense of a much lower loss in recall. Operationally, a higher precision translates to a lower rate of false alarms which is a desirable attribute for an algorithm to be used in a busy clinical setting. When evaluated on a higher risk patient cohort, the EF algorithm also offered very high recall performance, which translates operationally to a very low rate of missed high risk patients, another desirable attribute for the chosen clinical environment. The study explored various prediction horizons that would allow clinicians to evaluate a patient’s risk of deterioration 2–8 h before an actual event. Time windows are aligned with workflows and clinical and bedside planning activities and are chosen to have high implementable value. These were chosen to fit with bedside workflows and are a balance between the clinical urgency and dynamic resource allocations during a clinical shift, and the technical constraints of the ability to model event rates. 8 h is the length of a typical shift for nursing staff, and there are three such windows in a day. Thus an 8 h window would have visibility for the next shift of possible arising problems with deteriorating patients. 4 h is half a shift but is also approximately the time period of a medical session (morning afternoon or evening) which is the workflow duration for an outpatient clinic, or a theatre session. This means that the horizon of interest is approximately the duration of the next activity that would take the doctor off the wards with other clinical tasks. 1 h is too short, and few observations are to occur in the next hour, and even fewer if patients are not already sick (i.e. activating a medical response criteria). 2 h is the shortest useful time frame, where the event rate is common enough to plausibly be modelled. So, while we could have used 2.5 h, or 10 h or continuous scales or any arbitrary time window, it would have been only based on event rates and mathematical criteria. The challenge would be how to integrate these into practice.

The findings in recent systematic reviews show that the main focus of current approaches is to predict an incident of deterioration, such as, cardiac arrest, transfer to ICU, or death^1,2^. However, our approach of “predicting the alert”, that is, the output of an early warning system, is novel and can be distinguished from such algorithms in the literature. This approach is useful, in practice, as it directs allocation of resources to review, and some direction to what interventions would be directed towards. Though not directly comparable, we discuss current state of the art approaches in this domain, particularly efforts utilizing EMR data for predicting early deterioration in the context of acute critical illnesses such as sepsis, acute kidney injury (AKI) and acute lung injury (ALI)^14^, physiological deterioration after gastrointestinal cancer surgery^15^ and unplanned transfer to the ICU^3^. Lauritsen et al.^14^ reported results obtained for prediction window of 3, 6, 12 and 24 h ahead using laboratory data and vital signs. Although not directly comparable with our model, mean AUC-PRCs of 0.088, 0.068 and 0.134 were reported for a model predicting the risk of sepsis, AKI and ALI 6 h before their onset. These performance measure were remarkably lower to our model at 6 h prediction window before onset. The study presented in Clifton et al.^15^ was restricted to a postoperative ward at a cancer center with only 200 patients included in the study. Models were limited to four vital signs (SPO2, HR, BP and RR) and obtained partial AUC-ROC 0.28 for False positive Range FPR = [0, 0.15]. Kinipis et al.^3^ evaluated the performance of a regression model to predict the risk of an unplanned transfer to the ICU within the next 12 h (AUC 0.82, sensitivity 49). This study used laboratory tests, vital signs, neurological status checks, open source composite indices for severity of illness, longitudinal comorbidity burden end of life care directives and health care utilization services indicators (e.g. length of stay) as predictors. While employing similar data, with a common objective to identify and intervene early to prevent deterioration, we argue our approach offers the advantage of not having been developed on potentially confounding data (from a clinical setting where a different intervention strategy was likely used) and is easy to implement as it aligns with the protocols already mandated for use in the clinical setting.

Unlike work presented by Clifton et al.^15^ and Kinipis et al.^3^, our approach also incorporates explainability and provides clinicians with an insight into how the predicted deterioration is likely to manifest. This allows the clinical team to focus on the likely cause and react accordingly.

Figure 3 is an example of which influential features for an observation predicted a deterioration. In the context of the patient diagnosis, an awareness of how a patient deteriorates provides an important guide on how to pre-emptively address this and guide doctors on appropriate treatment options. The two cases shown in Fig. 4 illustrate individual interpretation. For Patient 1 (Fig. 4a), respiratory rate and O2 saturation were the features with greatest importance. For the Patient 2 (Fig. 4b), blood pressure and temperature were the features of importance. The first patient was, therefore, a greater risk of an emergency caused by respiratory failure, while the second patient was more likely to develop an emergency looking like a septic episode. Having this information several hours before it was identified by current protocols can give clinicians significant opportunity to deliver improved patient care and outcomes.

**Figure 5:**
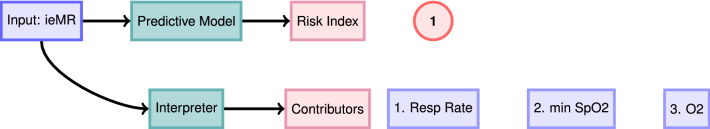
PRoD pipeline: With every appearance of a new observation in ieMR, data are fed into the model and the interpreter simultaneously. The Predictive Model is translated into Risk Index showed as a circle where colour and an associated number represents level of risk. The Interpreter provides a list of the top contributors in descending order explaining the prediction. Blue implies low values and red high values of the listed variable. With every new prediction dashboard is updated.

We have shown that data collected in EMRs can be leveraged to provide real-time decision support and support improved patient outcomes within the scope of existing mandated clinical protocols. The novel EF algorithm offers high precision and recall performance for the patient cohort of interest while identifying patients likely to deteriorate several hours before existing clinical protocols trigger. It also offers insight into factors likely to contribute to the deterioration. While developed on the BTF protocol, the approach is extendable to any clinical deterioration protocol. Further research is planned to assess the developed algorithm in a trial clinical setting. An illustration of the PRoD pipeline in the clinical settings for such a study is shown in Fig. 5. The current work is limited in that it is developed on data from a single hospital and employs a limited cross section of data collected in the clinical setting (i.e. vital signs and some demographic information). Future research is required to explore the inclusion of other real or near real-time data such as pathology test results and the generalizability to other hospitals and health care settings. Lastly, once the algorithm is implemented and shown to be effective in reducing alerts, any algorithm refinements on new data will be confounded. The current algorithm could be by using similar iEMR data from peer hospitals with similar casemix and workflow not participating in the trial.

## Methods

### Study and data description

The data for this study was sourced from a major metropolitan hospital in Queensland, Australia. The study was approved by the Metro South Hospital and Health Service Human Research Ethics Committee (HREC Ref No HREC/18/QPAH/525) and CSIRO Health and Medical Human Research Ethics Committee (CHMHREC Proposal 2019_015_RR). The study protocol including all methods was reviewed and approved by the Metro South Health Service Human Research Ethics Committee (HREC identifier HREC/18/QPAH/525). All methods were performed in accordance with the relevant guidelines and regulations. The data provided for this study had all formal identifiers removed and was reviewed by the Metro South Hospital and Health Service Human Research Ethics Committee (HREC Ref No HREC/18/QPAH/525) and CSIRO Health and Medical Human Research Ethics Committee (CHMHREC Proposal 2019_015_RR) as low or negligible risk and the requirement for informed consent by subjects or their guardians was waived.

**Figure 6:**
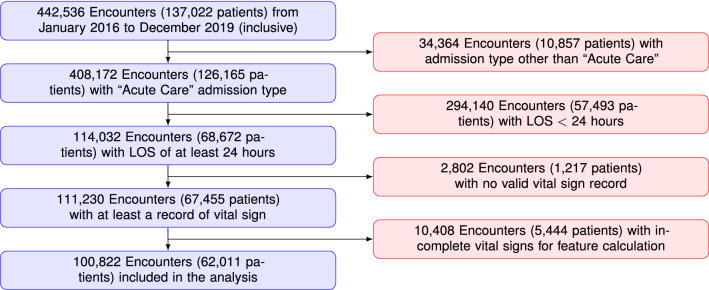
Cohort selection procedure.

Data were extracted from the Integrated Electronic Medical Record (ieMR) clinical data repository that provides access to near real-time patient information records. Ensuring availability of near real-time access to the candidate predictors helped ensure our future aim of deploying and trialing the developed algorithms for near real-time clinical decision support. All hospital patient encounters (defined as a hospital admission) from 1 January 2016 to 31 December 2019 were included along with their movements, treatments, and physiological and clinical conditions throughout their stay (e.g., wards admission, vital signs, pathology orders, medication orders, etc.). Figure 6 shows the inclusion/exclusion criteria for selecting the cohort of interest for this study.

In total, 442,536 hospital encounters (unique admissions) were collected throughout the four-year study period. Hospital encounters were restricted to “Acute Care” admission, given the nature of the target problem in this study (n=408,172). The encounters with admission types such as “Mental health care”, “Rehabilitation care”, “Maintenance Care” were excluded. Due to certain engineered features requiring an adequate number of observations to calculate a value (e.g. standard deviation), only encounters with a length of stay greater than 24 h were employed for model development and validation (LOS ≥ 24, n=114,032). The vital signs that were measured during the hospital stay were recorded and used for identifying risk of deterioration. As a result, only encounters with corresponding records of vital signs in our data (n=111,230) were retained. A final validation was performed to only include encounters where a BTFs alert (our response variable) could be calculated from available data. This resulted in a total of 100,822 hospital encounters from 62,011 patients over the study period to be included in the analysis.

### Modeling pipeline

#### Data for modeling and evaluation

Three years of data from January 2016 to December 2018 (inclusive) were used as the training and development sets. The final year (i.e., 2019) of data were held out as the test set. While the initial aim was to use the full 2019 calendar year as the testing period, exploratory analysis of the data revealed a change in vital sign data collection patterns from October 2019. Further investigation revealed that corresponded with a change in business processes at the hospital. This period was quarantined in consultation with clinical experts and data from January 2019 to September 2019 (inclusive) were used as the test set for this study.

#### Model features

The following seven vital signs typically used in EWS like BTF in clinical settings were employed as potential predictors (risk factors) in the developed model: Systolic and Diastolic Blood Pressure, Mean Arterial Pressure, Heart Rate, Temperature, Respiratory Rate, Oxygen Saturation (SpO2), and Oxygen flow rate (O2 flow rate). A measure of a patient’s level of consciousness (known as the AVPU scale) was also employed. The AVPU scale consists of 4 possible values: Alert, Verbal, Pain and Unresponsive patient, one of which is selected by a clinician when assessing a patient’s level of consciousness. Table 1 reports covariance balance analysis between these variables on training and test data. Trends and variations of the selected vital signs in a given historical time window were calculated and used as input features to the predictive models. Specifically, a historical window of 24 h was defined to collect observations and to monitor the stability of a patient’s condition. The measured values of all vital signs in any time window were analysed and the patterns of changes were quantified into a number of variables. The following features were calculated for each of the aforementioned vital signs (excluding O2 flow rate) in the past 24 h historical window: minimum, maximum, mean, median, standard deviation, frequency, number of measured vital signs (at the current observation date-time), number of overall observations in the historical 24-h window, last (most recent) valid value, interval in between the last two observations and slope between the two most recent observations. The *slope* feature was defined as the change of a given vital sign over its two most recent observations divided by the time interval between these observations, i.e., $Slope = (y_2-y_1)/(t_2-t_1)$, where $y_2$ is the current observation of a vital sign and $y_1$ is the previous observation, $t_2$ and $t_1$ are the corresponding timestamps for these two observations, respectively. Due to the incomplete nature of the O2 flow and AVPU vital signs, two binary flags for each of them were defined. An O2 Flow flag denoted if any O2 flow had been measured in the past 24 h, while an AVPU flag denoted if any AVPU status had been assigned in the past 24 h. Demographic information inclusing age, gender and the patients’ length of stay at the point of prediction were also considered as additional predictors. While selecting potential predictors, care was taken to avoid using information that was not available in real-time in the ieMR system. This ensured the feasibility of our model as a bedside decision support system.

#### Response variable

As we employed a “predict the alert” approach, the response variable was defined as whether any one of the seven vital sign measurements triggered a “between the flags” red alert. A red alert indicates the potential for a moderate or high risk derangement of a patient within existing clinical workflows in the Hospital.

#### Machine learning models

Clinical data often include a higher frequency of vital sign measurements from sicker patients due to the need for increased monitoring of such patients and an expected longer length of stay. We proposed an encounter-level sampling approach to guide bootstrapping wherein each bootstrapped sub-sample takes only one observation per unique encounter. This minimises potential biases towards observations from sicker patients and accounts for temporal correlation between observations from individual patient encounters. The bootstrap sub-sampling was also driven by inverse-probability factor to enforce even sampling between “no-deterioration-event” and “deterioration-event” observations.

**Figure 7:**
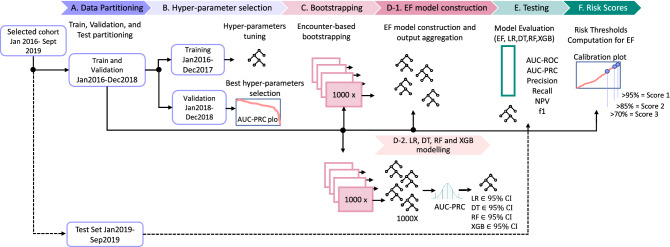
Detailed modeling procedure: (A) Once data are split on training and test, training data (Jan 2016–Dec 2018) are additionally partitioned on two subsets. (B) Model candidates (LR, RF, DT and XGB) are trained for specified hyper-parameter grid on data Jan 2016-Dec 2017 and evaluated with AUC-PRC on validation partition. The set of parameters producing the best AUC-PRC score is selected. (C) 1000 bootstrapped sub-samples are generated. (D-1 and D-2) 1000 individual LR, RF, DT and XGB models are trained along Encounter-based Forest-like (EF) ensemble model. (E) EF ensemble and selected LR, RF, DT, XGB models are evaluated on 1000 bootstrapped samples drawn with replacement from the test set. (F) Threshold for Risk index 1, 2 and 3 determined for selected EF model are computed based on the calibration curve.

The encounter-level bootstrapping was repeated 1000 times to obtain 1000 bootstrap sub-samples (Fig. 7, step C). Logistic regression using lasso regularisation (LR), Decision Trees (DT), Random Forest (RF) and XGBoost (XGB) were chosen as candidate machine learning algorithms (Fig. 7, step D-2). A novel forest-like ensemble, called an Encounter-based Forest (EF) (Fig. 7, step D-1), was also constructed featuring the chosen encounter-level bootstrapping, a decision tree architecture for each sub-sample, and bootstrap aggregation for obtaining the final probability predictions. The performance of this EF ensemble was also compared to the other chosen ML algorithms (Fig. 7, step E).

To find the best set of hyperparameters for all model candidates, we performed training on the first two years of the training data and evaluation of the AUC-PRC score on the remaining year of the training data, referred to as the validation partition. For each model candidate, we chose the set of hyperparameters that maximized AUC-PRC and refit it on whole training partition. All models were re-trained on each of 1000 generated encounter-based bootstrap sub-samples. The steps of the adopted approach are illustrated in Fig. 7, steps A, B.

#### Retrospective evaluation

Data split on training and test partition was performed following the TRIPOD recommendation for time series data. We evaluated the performance of the all considered models on a held-out portion of data kept for testing, dating from January 2019 up to October 2019. As our primary benchmark we compared algorithm predictions to the red alerts reported in the considered data extract. In line with the TRIPOD statement, model performance was assessed on the held-out test dataset (encounters dated Jan 2019 to September 2019) using the Area Under the Receiver Operator Characteristic Curve (AUC-ROC) as the measure of performance. Though AUC-ROC is popularly used to quantify the discriminative power of predictive models developed for clinical applications, research also indicates that it is a misleading performance indicator for imbalanced datatsets^16,17^ and may provide an inaccurate measure of targeted clinical utility as it assumes that positive and negative predictions are equally important and is insensitive to the dominance of a minor class, patients at risk of deterioration in our case. Hence, in addition to AUC-ROC, we also computed AUC-PRC which has been shown to be more informative than AUC-ROC^18^. Additionally, precision, recall, NPV and f1 score metrics are computed as they are more suitable for evaluation of model performance on imbalanced data. The discriminative power of the selected model for each susceptibility group was assessed by validating the predicted outputs for each susceptibility group individually against the corresponding ground-truth and computing the precision and recall.

#### Deterioration risk index computation

Assessment of the calibration curve was used to gain insight into the performance of the model when predicting a range of outcome probabilities. It was obtained by grouping predictions into quantiles (we used 10) which were then represented as points such that the proportion of true outcomes lies on the *x*-axis and mean predicted probability on the *y*-axis. We used the calibration curve to translate probabilistic output values of EF model into susceptibility groups 1, 2 and 3 (Risk Index 1, 2 and 3) by grouping the predicted probabilities into the top $5\%$ (i.e. predicted $p>0.95$), $15\%$ (i.e. predicted $p>0.85$) and $30\%$ i.e. predicted ($p>0.70$) risk levels based on the calibration curve analysis (Fig. 7, step F).

#### Explainability module

Explainability of predictions was the second aim of this study. By understanding the most influential feature changes that drive the deterioration, we are proposing the route or phenotype of clinical deterioration. Unlike transparent models such as regression models, which do not require an external model to interpret the association between the input and predicted output like logistic regression, ML models require an external explanability module. In the presented work, to evaluate the feature relevance and association to predicted outcome we adopted explainer method initially developed for tree-based models^19^, based on computing Shapley values adopted from game theory. This method calculates the marginal contribution of each feature by computing the difference between the predictive performance of a model with and without each feature included in the model. The level of relevancy of a single feature is obtained by averaging the marginal contribution of that feature across all feature subsets in which it was included. We constructed an explainer module for each individual model of the EF ensemble individually on training data. The prediction explanation for a tested sample was computed by averaging the relevance of features computed by explainer module constructed for every model individually across all 1000 models.

#### Statistical analysis

To assess covariate balance between the test and training sets, two-sample *t*-tests for normally distributed variables were employed and chi-square test of proportions were employed for categorical data. The *p* values reported in Table 1 are presented for descriptive proposes and not intended to assess statistical significance of the comparison. The Standardised Mean Difference (SMD) was calculated for continuous and discrete variables as proposed in^20^. Reported means and $95\%$ confidence intervals (CIs) of 1000 bootstrapped models obtained on test data were calculated by averaging and computing the CIs using the Student *t*-test. To evaluate selected population average models and EF we computed the mean for each metric using 1000 bootstrapped samples drawn with replacement from the test set. Each sub-sample had the same class distribution as the original test set. CIs are computed with two paired *t*-test. All analyses were performed using Python version 3.6 and R version 4.0.4. Scikit-Learn’s implementation of DT, RF, and XGB was employed in this study (version 0.23.2^21^).

## Supplementary Information

Supplementary Tables.

## Data Availability

The datasets analysed in the current study are not publicly available. Due to reasonable privacy and security concerns and requirements imposed by the ethics approval process, they are not redistributable to researchers other than those engaged in the ethics committee approved research protocol. Correspondence and requests for data should be addressed to A.B. (email: aida.brankovic@csiro.au).

## Code availability

In this work we used following open-source libraries to conduct our experiments: For development of machine learning model we used sklearn (https://scikit-learn.org/) library developed for Python programming language. Shaply values used for explanations of the model were calculated with SHAP package (https://github.com/slundberg/shap) . The analysis, training and tests were performed with custom code written in Python 3.6. Statistical analysis of model performance is conducted with scipy.stats Python module. All procedures and implementations are in Methods and supplement and allow identical replication. Any inquiry regarding the technical details of the specific models can be made by relevant parties to the corresponding author (email: aida.brankovic@csiro.au).
