# Author Correction: Explainable machine learning for real-time deterioration alert prediction to guide pre-emptive treatment

**DOI:** 10.1038/s41598-022-18011-3

**Published:** 2022-08-05

**Authors:** Aida Brankovic, Hamed Hassanzadeh, Norm Good, Kay Mann, Sankalp Khanna, Ahmad Abdel-Hafez, David Cook

**Affiliations:** 1 CSIRO Australian E-Health Research Centre, Brisbane, QLD 4029 Australia; 2 Metro South Health, Brisbane, QLD 4102 Australia; 3 Intensive Care Unit, Princess Alexandra Hospital, Brisbane, QLD 4102 Australia

Correction to: *Scientific Reports* https://doi.org/10.1038/s41598-022-15877-1, published online 11 July 2022

The original version of this Article contained an error in Figure 1, where the labels for ‘Flag %’ and ‘No Flag %’ were interchanged in the legend of the graphs. The original Figure 1 and accompanying legend appear below.

**Figure 1:**
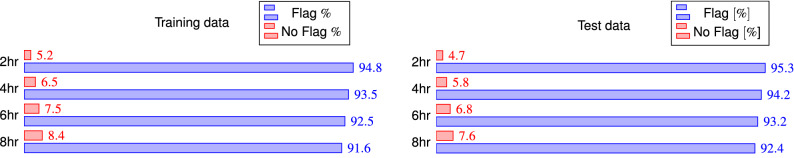
Class distribution per alerting horizon in train and test data partitions.

The original Article has been corrected.

