# Supplementary material for: Explainable machine learning for real-time deterioration alert prediction to guide pre-emptive treatment
Source: Sci Rep. 2022 Jul 11;12:11734. doi: 10.1038/s41598-022-15877-1 (PMC9273762; doi:10.1038/s41598-022-15877-1)
Supplement: Supplementary file 1 — Supplementary Tables. [file 41598_2022_15877_MOESM1_ESM.pdf]

Explainable machine learning for real-time deterioration alert prediction to guide pre-emptive treatment

Additional Information

Table S1 provides details of the search space for each hyperparameter and the final selected value.

	Hyper-parameter grid	Best parameter
**LR**	'C': [1e-05, 3.59381366e-05, 0.000129154967 0.000464158883, 0.00166810054, 0.0059948425, 0.0215443469, 0.0774263683, 0.27825594]	0.0215443469
**DT**	'max_depth': [None, np.arange(10,45,10)]	10
	'min_samples_split': [2, 50, 500, 10000]	500
	'class_weight': ['balanced', None]	None
**XGB**	'max_depth': [6, 10 ,15]	10
	'min_child_weight': [1, 500, 1000, 10000]	1
	'eta': [0.05, 0.1, 0.3]	0.05
	'subsample':[0.7, 1]	1
	'objective':['binary:logistic']	binary:logistic
	'eval_metric':['aucpr']	aucpr
**RF**	'n_estimators': [100, 150, 200]	150
	'max_features': ['auto']	auto
	'max_depth': [15, 35, None]	None
	'min_samples_split': [2, 1000, 10000]	2
	'bootstrap': [True]	True
	'class_weight':[None]	None

Table S1. Parameter grid used for the optimization of hyper-parameters

Table S2 provides detailed results of different ML models and prediction windows.

Model	AUC-ROC [95% CI]	Brier score [95% CI]	NPV [95% CI]	Precision [95% CI]	Recall [95% CI]	F1 [95% CI]	AUC-PRC [95% CI]
**LR**							
2hr	0.859 [0.859 0.86]	0.041 [0.04 0.041]	0.971 [0.971 0.971]	0.49 [0.489 0.491]	0.395 [0.394 0.395]	0.437 [0.437 0.438]	0.405 [0.404 0.405]
4hr	0.837 [0.837 0.838]	0.047 [0.047 0.047]	0.958 [0.958 0.958]	0.537 [0.536 0.538]	0.309 [0.308 0.309]	0.392 [0.392 0.392]	0.392 [0.391 0.393]
6hr	0.824 [0.824 0.824]	0.053 [0.053 0.053]	0.948 [0.948 0.948]	0.576 [0.575 0.578]	0.253 [0.252 0.253]	0.351 [0.351 0.352]	0.39 [0.389 0.391]
8hr	0.813 [0.813 0.813]	0.058 [0.058 0.058]	0.939 [0.939 0.939]	0.596 [0.594 0.597]	0.22 [0.219 0.22]	0.321 [0.32 0.321]	0.387 [0.386 0.388]
**DT**							
2hr	0.857 [0.857 0.858]	0.032 [0.032 0.032]	0.979 [0.979 0.979]	0.75 [0.746 0.755]	0.556 [0.556 0.557]	0.637 [0.636 0.638]	0.562 [0.561 0.562]
4hr	0.814 [0.814 0.815]	0.044 [0.043 0.044]	0.968 [0.968 0.968]	0.726 [0.722 0.73]	0.464 [0.463 0.465]	0.564 [0.563 0.565]	0.505 [0.505 0.506]
6hr	0.793 [0.793 0.793]	0.052 [0.052 0.052]	0.958 [0.958 0.958]	0.718 [0.713 0.723]	0.405 [0.404 0.406]	0.516 [0.515 0.516]	0.477 [0.476 0.477]
8hr	0.781 [0.781 0.782]	0.058 [0.058 0.058]	0.95 [0.95 0.95]	0.698 [0.693 0.704]	0.37 [0.369 0.371]	0.481 [0.48 0.481]	0.459 [0.459 0.46]
**RF**							
2hr	0.904 [0.904 0.904]	0.036 [0.036 0.036]	0.98 [0.98 0.98]	0.719 [0.718 0.719]	0.586 [0.586 0.586]	0.645 [0.645 0.646]	0.632 [0.632 0.633]
4hr	0.873 [0.873 0.874]	0.047 [0.047 0.047]	0.969 [0.969 0.969]	0.703 [0.702 0.704]	0.497 [0.496 0.497]	0.582 [0.582 0.582]	0.577 [0.577 0.578]
6hr	0.854 [0.854 0.854]	0.054 [0.054 0.055]	0.96 [0.96 0.96]	0.705 [0.704 0.706]	0.443 [0.442 0.443]	0.544 [0.544 0.544]	0.55 [0.55 0.551]
8hr	0.841 [0.841 0.841]	0.06 [0.06 0.06]	0.953 [0.953 0.953]	0.712 [0.711 0.713]	0.408 [0.408 0.409]	0.519 [0.519 0.519]	0.536 [0.536 0.537]
**XGB**							
2hr	0.9 [0.899 0.9]	0.032 [0.032 0.032]	0.98 [0.98 0.98]	0.622 [0.62 0.623]	0.596 [0.595 0.596]	0.608 [0.608 0.609]	0.628 [0.628 0.629]
4hr	0.864 [0.864 0.864]	0.043 [0.043 0.043]	0.97 [0.97 0.97]	0.601 [0.6 0.602]	0.511 [0.511 0.512]	0.552 [0.552 0.553]	0.569 [0.569 0.569]
6hr	0.84 [0.84 0.84]	0.051 [0.051 0.051]	0.961 [0.961 0.961]	0.599 [0.598 0.601]	0.458 [0.458 0.459]	0.519 [0.519 0.52]	0.537 [0.537 0.537]
8hr	0.825 [0.825 0.825]	0.057 [0.057 0.057]	0.954 [0.954 0.954]	0.6 [0.599 0.601]	0.426 [0.426 0.427]	0.498 [0.498 0.498]	0.519 [0.519 0.52]

Table S2. Average performance metrics and corresponding 95% CI obtained on test data (January - September 2019) of ML models trained on 1000 bootstrapped subsets of the original training partition across the time windows of 2, 4, 6 and 8 hours

Table S3 reports performance metrics obtained for EF and selected DT, RF, XGB models with 95% CI using 1000 bootstrapped samples drawn with replacement from the test set.

**Model** Time	**AUC-ROC** [95% CI]	**Brier score** [95% CI]	**NPV** [95% CI]	**Precision** [95% CI]	**Recall** [95% CI]	**F1** [95% CI]	**AUC-PRC** [95% CI]
**LR**							
2hr	0.8593 [0.8592 0.8593]	0.0402 [0.0402 0.0403]	0.9703 [0.9703 0.9703]	0.4945 [0.4943 0.4948]	0.3863 [0.3862 0.3865]	0.4338 [0.4336 0.434]	0.4048 [0.4046 0.405]
4hr	0.8372 [0.8371 0.8372]	0.0469 [0.0469 0.0469]	0.9585 [0.9585 0.9585]	0.5397 [0.5395 0.5399]	0.3102 [0.3101 0.3104]	0.394 [0.3938 0.3941]	0.3927 [0.3926 0.3929]
6hr	0.8233 [0.8233 0.8234]	0.053 [0.053 0.053]	0.9475 [0.9474 0.9475]	0.5773 [0.577 0.5775]	0.2509 [0.2508 0.251]	0.3498 [0.3496 0.3499]	0.3893 [0.3891 0.3894]
8hr	0.8112 [0.8112 0.8113]	0.0583 [0.0583 0.0583]	0.9386 [0.9386 0.9386]	0.5949 [0.5947 0.5952]	0.2138 [0.2137 0.214]	0.3146 [0.3144 0.3147]	0.3837 [0.3836 0.3839]
**DT**							
2hr	0.8423 [0.8422 0.8424]	0.0315 [0.0315 0.0315]	0.9777 [0.9777 0.9777]	0.8561 [0.8559 0.8563]	0.5351 [0.5349 0.5352]	0.6585 [0.6584 0.6587]	0.5919 [0.5917 0.5921]
4hr	0.8151 [0.815 0.8152]	0.0425 [0.0425 0.0425]	0.9672 [0.9671 0.9672]	0.7962 [0.796 0.7964]	0.4541 [0.4539 0.4543]	0.5783 [0.5782 0.5785]	0.5329 [0.5327 0.5331]
6hr	0.7941 [0.794 0.7942]	0.0528 [0.0528 0.0528]	0.9584 [0.9584 0.9584]	0.6603 [0.6601 0.6604]	0.4151 [0.415 0.4153]	0.5098 [0.5096 0.5099]	0.4674 [0.4672 0.4677]
8hr	0.7872 [0.7871 0.7873]	0.0584 [0.0584 0.0584]	0.9505 [0.9505 0.9505]	0.6432 [0.643 0.6434]	0.3774 [0.3772 0.3775]	0.4757 [0.4755 0.4758]	0.4727 [0.4726 0.4729]
**XGB**							
2hr	0.9009 [0.9008 0.9009]	0.0324 [0.0324 0.0324]	0.9801 [0.9801 0.9802]	0.6159 [0.6157 0.6161]	0.5932 [0.593 0.5934]	0.6043 [0.6042 0.6045]	0.631 [0.6308 0.6312]
4hr	0.8652 [0.8651 0.8652]	0.0423 [0.0423 0.0424]	0.9697 [0.9697 0.9697]	0.608 [0.6078 0.6082]	0.5041 [0.5039 0.5042]	0.5512 [0.551 0.5513]	0.5719 [0.5718 0.5721]
6hr	0.8396 [0.8395 0.8397]	0.0502 [0.0502 0.0502]	0.9609 [0.9609 0.9609]	0.6116 [0.6114 0.6117]	0.4551 [0.455 0.4553]	0.5219 [0.5217 0.522]	0.5406 [0.5405 0.5408]
8hr	0.8264 [0.8264 0.8265]	0.0555 [0.0555 0.0556]	0.9537 [0.9537 0.9537]	0.6102 [0.61 0.6104]	0.423 [0.4228 0.4231]	0.4996 [0.4995 0.4998]	0.5222 [0.522 0.5223]
**RF**							
2hr	0.9039 [0.9038 0.9039]	0.0372 [0.0372 0.0372]	0.98 [0.98 0.98]	0.7121 [0.7119 0.7123]	0.5867 [0.5865 0.5869]	0.6433 [0.6432 0.6435]	0.6362 [0.636 0.6364]
4hr	0.8734 [0.8733 0.8734]	0.0463 [0.0463 0.0463]	0.9689 [0.9689 0.9689]	0.7308 [0.7307 0.731]	0.4866 [0.4864 0.4867]	0.5842 [0.584 0.5843]	0.5816 [0.5814 0.5817]
6hr	0.8551 [0.855 0.8552]	0.053 [0.053 0.053]	0.9603 [0.9603 0.9603]	0.7061 [0.706 0.7063]	0.4414 [0.4412 0.4416]	0.5432 [0.5431 0.5434]	0.5555 [0.5553 0.5557]
8hr	0.8404 [0.8403 0.8405]	0.0595 [0.0595 0.0595]	0.9529 [0.9529 0.9529]	0.7288 [0.7286 0.7289]	0.4068 [0.4066 0.4069]	0.5221 [0.522 0.5223]	0.542 [0.5418 0.5421]
**EF**							
2hr	0.8793 [0.8792 0.8794]	0.0292 [0.0292 0.0292]	0.9784 [0.9784 0.9784]	0.8149 [0.8147 0.8151]	0.5506 [0.5505 0.5508]	0.6572 [0.657 0.6574]	0.6179 [0.6177 0.6181]
4hr	0.8387 [0.8387 0.8388]	0.0398 [0.0398 0.0398]	0.9674 [0.9674 0.9674]	0.7994 [0.7992 0.7996]	0.458 [0.4579 0.4582]	0.5824 [0.5822 0.5825]	0.5609 [0.5608 0.5611]
6hr	0.8203 [0.8202 0.8204]	0.0476 [0.0475 0.0476]	0.9572 [0.9572 0.9572]	0.8442 [0.844 0.8444]	0.391 [0.3908 0.3911]	0.5344 [0.5343 0.5346]	0.5373 [0.5371 0.5374]
8hr	0.8091 [0.809 0.8092]	0.0535 [0.0535 0.0535]	0.9492 [0.9492 0.9493]	0.8521 [0.8519 0.8522]	0.353 [0.3529 0.3532]	0.4992 [0.4991 0.4994]	0.5262 [0.526 0.5263]

Table S3. Performance metrics computed for EF and selected DT, RF, XGB models with 95% CI on the test set for Jan 2019-Sept 2019 across the prediction window of 2, 4, 6 and 8 hours
